# Intraoperative echocardiographic indicator for optimal bilateral pulmonary artery banding

**DOI:** 10.1007/s11748-025-02156-9

**Published:** 2025-05-14

**Authors:** Tetsuri Takei, Yukihiro Kaneko, Ryoichi Kondo, Naho Morisaki, Ikuya Achiwa

**Affiliations:** 1 Division of Cardiovascular Surgery, National Center for Child Health and Development, Tokyo, Japan; 2 Kitatama Hospital, 4-1-1, Chofugaoka, Chofu, Tokyo 182-0021 Japan; 3 Division of Cardiovascular Surgery, Kitazato University Hospital, Sagamihara, Kanagawa Japan; 4 Department of Social Medicine, National Center for Child Health and Development, Tokyo, Japan; 5 Isseido Clinic, Tokyo, Japan

**Keywords:** Bilateral pulmonary artery banding, Congenital heart disease, Palliative cardiac surgery, Systemic–to–pulmonary blood flow ratio, Intraoperative echocardiography

## Abstract

**Background:**

We aimed to establish the most predictive echocardiographic indicator of appropriate tightness of bilateral pulmonary artery banding (BPAB).

**Methods:**

In part A of the study, we retrospectively analyzed the peak flow velocity (PV) and nadir flow velocity (NV) across the band and the ratio of NV to PV (velocity ratio: VR) to determine appropriate band tightness. In part B, we prospectively studied the utility of the best predictive indicators.

**Results:**

Thirty-one patients undergoing BPAB were enrolled in part A and identified as having appropriate pulmonary blood flow (APF), high pulmonary blood flow (HPF), or low pulmonary blood flow (LPF) during the postoperative period. The areas under the receiver operating characteristic curve (AUC) for HPF were 0.92 for PV, 0.99 for NV, and 0.99 for VR; the velocity thresholds were 2.47, 1.15, and 0.45 m/sec, respectively. For LPF, the AUCs were 0.63 for PV, 0.78 for NV, and 0.81 for VR, and the velocity thresholds were 2.70, 1.59, and 0.58 m/sec, respectively; thus, VR best indicated band tightness. In part B, we performed BPAB in 34 patients, adjusting the bands to achieve VRs between 0.45 and 0.58. The prevalence of HPF was significantly lower in part B than in part A, whereas those of LPF did not differ.

**Conclusion:**

In BPAB, we consider the optimal range of VR at banding site is between 0.45 and 0.58.

## Introduction

Bilateral pulmonary artery banding (BPAB) is an effective and less invasive palliative procedure performed in patients with congenital heart diseases that cause duct-dependent circulation. The success of BPAB depends on achieving an appropriate balance between systemic blood flow and pulmonary blood flow.

Criteria for determining the appropriate tightness of BPAB have not yet been standardized. In this study, we sought the most reliable echocardiographic indicator for appropriate banding tightness in retrospective and prospective investigations.

## Patients and methods

This study was approved by the Institutional Review Board of the National Center for Child Health and Development on March 30, 2018 (approval number 1781). Patients’ guardians provided informed written consent for our surgery, study, and publication of the data.

This study consisted of two parts. Part A was a retrospective investigation to identify the best predictive indicators of band tightness. Part B was a prospective investigation of the clinical efficacy of the best predictive indicators.

### Measurement of candidate predictive indicators

**Fig. 1:**
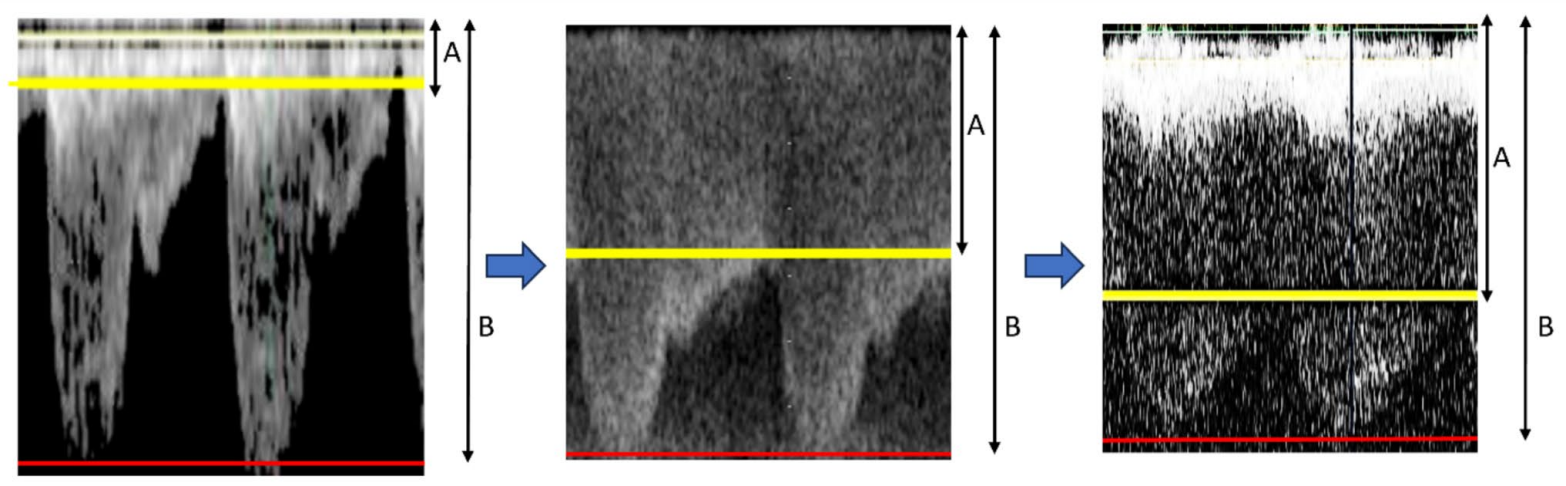
Intraoperative continuous-wave Doppler echocardiographic images across banding site of pulmonary artery. **A** (from top to yellow line) is nadir flow velocity. **B** (from top to red line) is peak flow velocity. Velocity ratio (**A** divided by **B**) increases with tighter banding. At the appropriate band tightness, the echocardiographic flow image depicts excessive pulmonary blood flow (left image), appropriate pulmonary blood flow pattern (middle image), or insufficient pulmonary blood flow (right image)

In our previous experience, while the pulmonary artery band was gradually tightened during surgery, echocardiography showed increases in not only systolic but also diastolic blood flow velocities. Therefore, we hypothesize that the increments of change in nadir flow velocity (NV) at diastole are greater than those in peak flow velocity (PV) at systole and that the ratio of nadir flow velocity to PV across the bands could serve as an indicator of band tightness. We arbitrarily selected three indicators to study: PV, NV, and ratio of NV to PV (velocity ratio [VR]) across the band. (Fig. 1) When measurements of both the right and left pulmonary arteries were available, we used the average of the two.

Change in arterial oxygen saturation (SaO_2_) after banding was also analyzed as an additional candidate indicator.

### Definition of the endpoints

Patients with appropriate pulmonary blood flow (APF) were hemodynamically stable after BPAB and proceeded to the next surgery in stable condition without requiring reintervention to regulate pulmonary blood flow. Patients with high pulmonary blood flow (HPF) had symptoms of heart failure; some died, some required tightening of the bands, and some needed supplemental nitrogen inhalation therapy. Patients with low pulmonary blood flow (LPF) had hypoxia and consequently died or required reintervention to increase pulmonary blood flow regulation.

### Patient groups

From April 2010 to May 2021, 68 patients underwent BPAB as initial surgery in our hospital (Tables 1 and 2). All these patients had duct-dependent circulation, truncus arteriosus, or aortopulmonary window. Some had heart diseases for which biventricular repair was suitable.

**Table 1:** Patient characteristics in part A

Patient no	Diagnosis	Ventricle repair type	At time of operation		SaO_2_ after band tightening (%)
			Age (days)	Weight (kg)	
Appropriate pulmonary blood flow					
1	HLHS (MA/AA)	Univentricular	2	3.1	84.6
2	HLHS (MS/AS)	Univentricular	3	2.6	92.3
3	Truncus arteriosus	Biventricular	1	2.62	75.2
4	Aortopulmonary window	Biventricular	2	2.3	87.6
5	Interrupted aortic arch type B	Biventricular	6	2.43	92.1
6	SV, PA	Univentricular	6	2.9	87.9
7	SV, CoA	Biventricular	7	3.2	83.5
8	PA/IVS	Biventricular	15	2.9	71.6
9	Truncus arteriosus	Biventricular	3	2.98	82.5
10	HLHS (MS/AS)	Univentricular	6	2.06	91.6
11	Interrupted aortic arch type B	Biventricular	4	3.07	88.7
12	HLHS (MA/AA)	Univentricular	1	2.71	84.5
13	TAPVC, CoA	Biventricular	1	2.15	97.9
14	HLHS (MA/AA)	Univentricular	6	2.15	81.0
15	HLHS	Univentricular	3	3.24	87.1
16	HLHS (MA/AA)	Univentricular	4	2.15	92.4
17	CoA/VSD	Biventricular	5	1.91	85.5
18	PA/IVS	Univentricular	7	2.91	83.1
19	SV, interrupted aortic arch type A	Univentricular	38	4.32	59.9
20	CoA	Biventricular	2	3.15	98.1
High pulmonary blood flow					
21	PA/IVS	Biventricular	7	1.97	95.4
22	SV, AS	Univentricular	3	2.3	84.0
23	HLHS	Univentricular	1	2.1	62.8
24	HLHS (MA/AA)	Univentricular	2	1.58	79.6
Low pulmonary blood flow					
25	HLHS (MA/AA)	Univentricular	2	2.8	79.7
26	SV, AA	Univentricular	4	2.6	89.5
27	HLHS (MA/AA)	Univentricular	3	2.6	77.5
28	Interrupted aortic arch type A	Biventricular	12	4.1	73.5
29	SV, CoA	Univentricular	7	2.61	84.0
30	SV, interrupted aortic arch	Univentricular	3	2.7	80.7
31	SV, AS	Univentricular	1	3.2	81.0

*AA* aortic atresia, *AS* aortic stenosis, *CoA* coarctation of aorta, *HLHS* hypoplastic left heart syndrome, *MA* mitral atresia, *MS* mitral stenosis, *PA* pulmonary atresia, *PA/IVS* pulmonary atresia with intact ventricular septum, *SV* single ventricle, *TAPVC* total anomalous pulmonary venous connection, *VSD* ventricular septal defect

**Table 2:** Patient’s characteristics in part B

Patient no	Diagnosis	Ventricle repair type	At time of operation		Velocity ratio	
			Age (days)	Weight (kg)	Right	Left
Appropriate pulmonary blood flow						
1	PS/IVS	Biventricular	4	2.75	0.74	0.69
2	Interrupted aortic arch type B	Biventricular	2	2.08	0.65	0.66
3	HLHS (MS/AA)	Univentricular	3	3.05	0.68	0.67
4	HLHS (MS/AS)	Univentricular	3	2.65	0.64	0.62
5	HLHS	Univentricular	22	1.98	0.56	0.48
6	Truncus arteriosus	Biventricular	3	2.71	0.65	0.75
7	HLHS (MA/AA)	Univentricular	4	2.38	0.50	0.57
8	Aortopulmonary window	Biventricular	11	0.81	0.59	0.45
9	HLHS (MS/AS)	Univentricular	1	1.3	0.60	0.59
10	HLHS (MA/AS)	Univentricular	1	2.45	0.63	0.58
11	HLHS	Univentricular	2	2.12	0.59	0.62
12	Interrupted aortic arch type B	Univentricular	2	2.24	0.61	0.50
13	CoA	Biventricular	4	2.8	0.54	0.54
14	Aortopulmonary window	Biventricular	6	2.25	0.53	0.55
15	HLHS	Univentricular	3	2.58	0.50	0.50
16	Interrupted aortic arch type A	Biventricular	4	1.08	0.55	0.55
17	TAPVC, PA	Biventricular	63	4.1	0.58	0.54
18	SV, CoA	Univentricular	4	2.42	0.59	0.58
19	Interrupted aortic arch type A	Biventricular	2	1.93	0.52	0.54
20	Interrupted aortic arch type B	Biventricular	3	2.88	0.50	0.50
21	HLHS (MS/AS)	Univentricular	2	1.77	0.60	0.60
22	Interrupted aortic arch type BB	Biventricular	3	2.2	0.59	0.56
23	SV, PA	Univentricular	7	1.17	0.50	0.50
24	CoA/VSD	Biventricular	4	2.81	0.46	0.46
25	SV, PA	Univentricular	4	2.1	0.45	0.45
26	SV, PA	Univentricular	3	1.6	0.56	0.50
Low pulmonary blood flow						
27	Truncus arteriosus	Biventricular	3	2.6	0.53	0.56
28	SV, PA	Univentricular	5	2.29	0.58	0.63
29	DORV, remote VSD, AS	Univentricular	1	3.14	0.57	0.48
30	HLHS (MS/AS)	Univentricular	1	3.03	0.60	0.60
31	Interrupted aortic arch type B	Biventricular	2	2.79	0.50	0.50
32	SV, CoA	Univentricular	5	2.64	0.59	0.63
33	SV, CoA	Univentricular	5	2.8	0.66	0.67
34	Truncus arteriosus	Biventricular	3	2.77	0.56	0.46

*AA* aortic atresia, *AS* aortic stenosis, *CoA* coarctation of aorta, *DORV* double-outlet right ventricle, *HLHS* hypoplastic left heart syndrome, *MA* mitral atresia, *MS* mitral stenosis, *PA* pulmonary atresia, *PA/IVS* pulmonary atresia with intact ventricular septum, *PS/IVS* pulmonary stenosis with intact ventricular septum, *SV* single ventricle, *TAPVC* total anomalous pulmonary venous connection, *VR* velocity ratio, *VSD* ventricular septal defect

Of the 68 patients who underwent BPAB, 65 were included in this study. We excluded 2 patients who exhibited low output syndrome regardless of band tightness, which necessitated extracorporeal membrane oxygenation support, and one patient who did not undergo the next surgery because of arrhythmia that was probably caused by a genetic disorder.

Thirty-one patients were enrolled in part A from April 2010 to June 2015. They were classified as having APF, HPF, or LPF according to their postoperative courses. We determined the optimal cutoff values of PV, NV, and VR at the banding sites. We also measured SaO_2_ after banding (Table 1).

Part B included the other 34 patients, who underwent BPAB between July 2015 and May 2021. In this study, we adjusted the bands according to the most predictive indicator determined in part A and evaluated the usefulness of the indicator postoperatively (Table 2).

### Surgical procedure

Our surgical technique for BPAB has been described elsewhere [1]. In part A, tightness of the bands was determined by the surgeons. To measure the most appropriate indicator in part B, we performed epicardial continuous-wave Doppler echocardiography with the EPIQ 7 Ultrasound System (Philips, Amsterdam, The Netherlands) and the X5-1 transducer, and adjusted to stay within the range determined in part A. The surgeons could complete the surgery without obtaining the targeted range of the indicator if they believed that a subjective decision would lead to better results.

### Statistical analysis

#### Part A

First, we used the Kruskal–Wallis test and the Mann–Whitney *U* test with the Bonferroni correction to compare postoperative PV, NV, VR, and SaO_2_ among the patients with different pulmonary blood flow measurements. For patients with multiple measurements obtained on postoperative days 0–7, we used the average of all measurements. We found that postoperative day was positively correlated with PV and NV; therefore, we repeated our analysis with predicted measurements of PV and NV on postoperative day 0 for each patient, using the value of *Y* at *X* = 0 in the *XY* axis, derived from models of all measurements and with patient-specific slopes to model linear increases in blood flow velocity over time.

Next, to evaluate the prognostic utility of postoperative PV, NV, VR, and SaO_2_, we generated receiver operating characteristic (ROC) curves using logistic regression, from which the area under the curve (AUC) and the threshold with optimal sensitivity and specificity were determined for each measurement. In conducting this analysis, we considered both LPF and HPF as outcomes.

#### Part B

We used Fisher’s exact test and the Mann–Whitney *U* test to compare parts A and B with regard to patients’ background characteristics, the measurements obtained, and prognosis.

For all descriptive and statistical analyses, we used Stata version 13 (StataCorp, College Station, TX, USA). *P* values of < 0.05 indicated statistical significance, and all statistical tests were two-tailed.

## Results

### Part A

Patients’ characteristics and surgical results are shown in Tables 1 and 3. At least one postoperative echocardiographic study was performed before postoperative day 7.

**Table 3:** Statistical analysis of results of parts A and B

Characteristic	Part A (*n* = 31)	Part B (*n* = 34)	*P* value
Age (days) [median (25%, 75%)]	3.5 (2, 6)	3 (2, 4)	0.6273
Weight (kg) [median (25%, 75%)]	2.6 (2.2, 3.1)	2.4 (2.1, 2.8)	0.0642
Type of ventricular repair			0.802
Biventricular	12 (38.7%)	15 (44.1%)	
Univentricular	19 (61.2%)	19 (55.6%)	
Outcome of patients			
Low pulmonary blood flow	7 (23%)	8 (24%)	
Appropriate pulmonary blood flow	20 (65%)	26 (76%)	
High pulmonary blood flow	4 (13%)	0 (0%)	

Age, weight, and plan of repair in parts A and B did not differ significantly. Rates of appropriate pulmonary blood flow (APF) and high pulmonary blood flow differed significantly (*P* = 0.046), but those of APF and low pulmonary blood flow did not (*P* = 0.531)

**Fig. 2:**
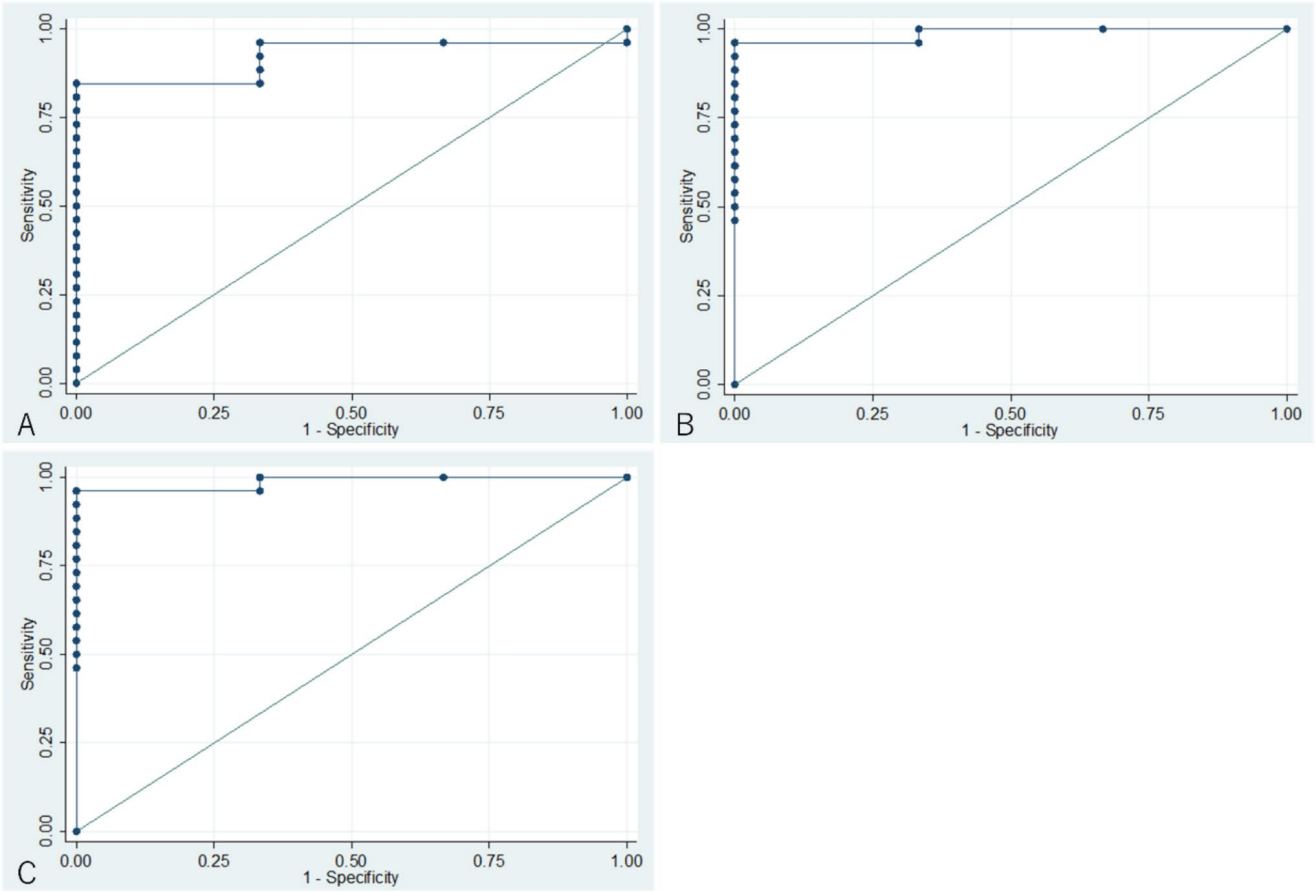
Prognostic utility of postoperative flow velocity on predicting high pulmonary blood flow. **A**: Peak flow velocity. The area under the receiver operating characteristic curve (AUC) is 0.92 (95% confidence interval [CI] 0.81–1.00). The threshold with the highest sensitivity and specificity is 2.47 m/s. **B**: Nadir flow velocity. The AUC is 0.99 (95% CI 0.95–1.00). The threshold with the highest sensitivity and specificity is 1.15 m/s. **C**: Velocity ratio. The AUC is 0.99 (95% CI 0.95–1.00). The threshold with the highest sensitivity and specificity is 0.45

The prognostic utility of predicted flow velocity measurements at the time of surgery to predict HPF is illustrated in Fig. 2. The AUCs were 0.92 for PV (95% confidence interval [CI] 0.81–1.00), 0.99 for NV (95% CI 0.95–1.00), and 0.99 for VR (95% CI 0.95–1.00). All three measurements had good prognostic value for HPF, especially NV and VR. The thresholds with the best sensitivity and specificity were 2.47 m/sec for PV, 1.15 m/sec for NV, and 0.45 for VR.

**Fig. 3:**
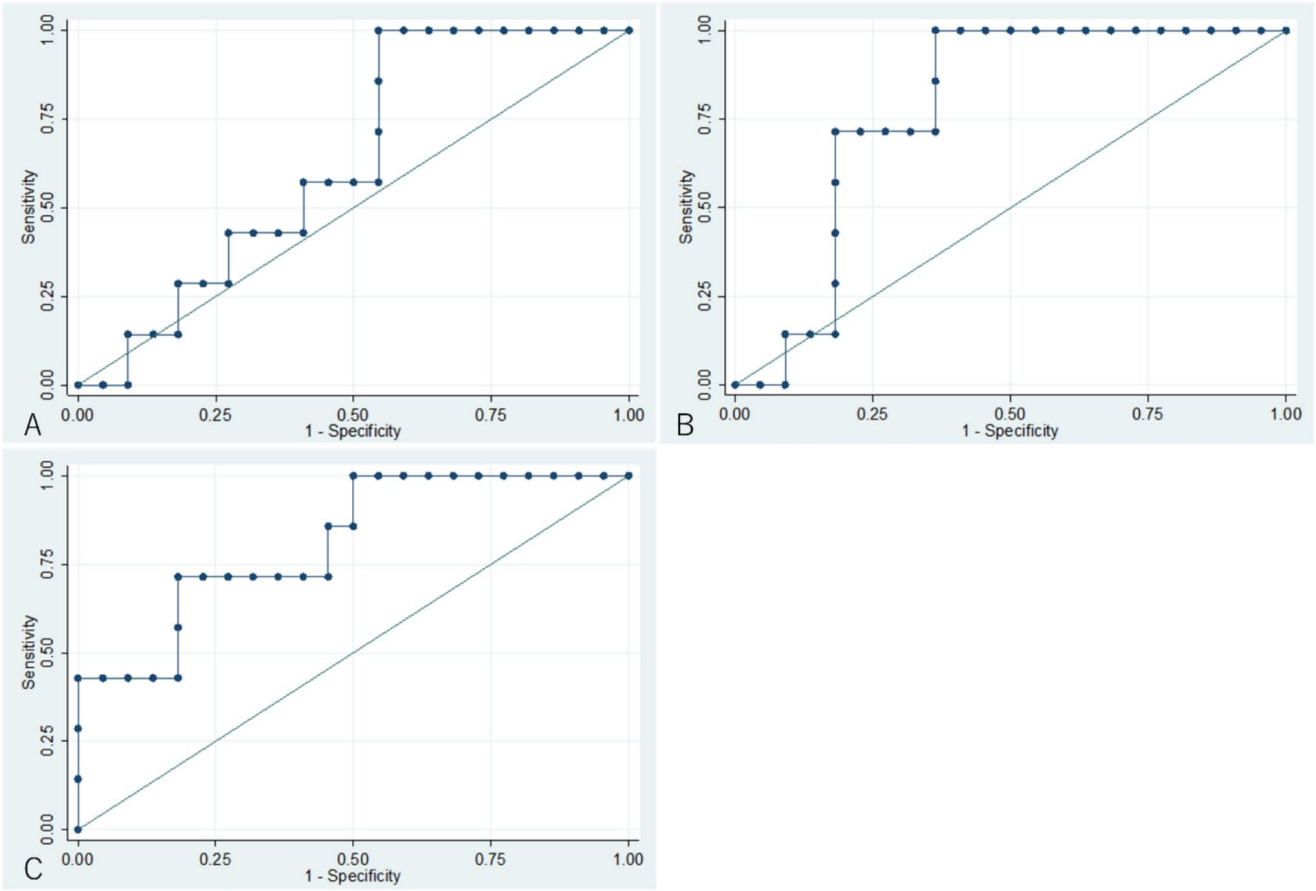
Prognostic utility of postoperative flow velocity in predicting low pulmonary blood flow. **A**: Peak flow velocity. The area under the receiver operating characteristic curve (AUC) is 0.63 (95% confidence interval [CI] 0.42–0.84). The threshold with the highest sensitivity and specificity is 2.70 m/s. **B**: Nadir flow velocity. The AUC is 0.78 (95% CI 0.61–0.95). The threshold with the highest sensitivity and specificity is 1.59 m/s. **C**: velocity ratio. The AUC is 0.81 (95% CI 0.63–0.96). The threshold with the highest sensitivity and specificity is 0.58

The prognostic utility of predicted flow velocity measurements at the time of surgery to predict LPF is illustrated in Fig. 3. The AUCs were 0.63 for PV (95% CI 0.42–0.84), 0.78 for NV (95% CI 0.61–0.95), and 0.81 for VR (95% CI 0.63–0.96). All measurements, particularly VR, had acceptable prognostic value for LPF. Thresholds with best sensitivity and specificity were 2.70 m/sec for PV, 1.59 m/sec for NV, and 0.58 for VR.

**Fig. 4:**
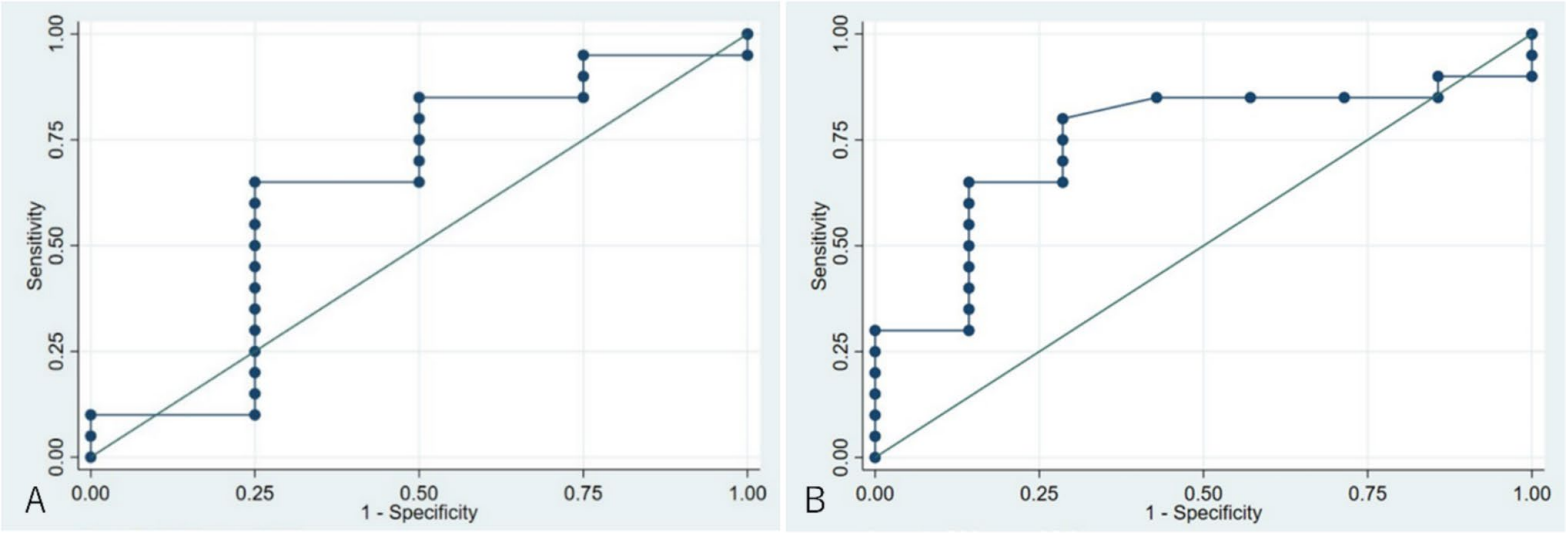
Intraoperative arterial oxygen saturation (SaO_2_) in part A. **A**: Comparison of SaO_2_ in patients with high pulmonary blood flow with that of patients with appropriate pulmonary blood flow (APF). The area under the receiver operating characteristic curve (AUC) is 0.64 (95% confidence interval [CI] 0.25–1.00). **B**: Comparison of SaO_2_ in patients with low pulmonary blood flow with that of patients with APF. The AUC is 0.75 (95% CI 0.54–0.96)

The ROC curve of SaO_2_ is shown in Fig. 4. The AUCs for HPF and LPF were 0.64 and 0.75, respectively.

### Part B

The results of part A indicated that VR was the best predictive indicator of band tightness. In part B, we adjusted band tightness with the aim of obtaining VRs between 0.45 and 0.58.

Patients’ characteristics are shown in Table 2 and 3. Data from two patients were excluded from analysis: one with Ebstein’s disease who required postoperative extracorporeal membrane oxygenation and one with a single ventricle who died of postoperative arrhythmia thought to be caused by inherent disease.

Of the 34 patients, 26 had APF, none had HPF, and 8 had LPF. In 17 patients, surgery finished with VR higher than the upper limit of the recommended range obtained in part A due to intraoperative the surgeons discretion for favorable outcome. The difference between the rate of HPF in part A and that in part B was significant (*P* = 0.046). The rates of LPF did not differ significantly (*P* = 0.531).

Twenty five patients with APF reached the final stage operation (total cavopulmonary connection or biventricular repair). One of the APF patients remains bidirectional Glenn circulation due to a genetic disease. Six of the LPF patients reached the final stage operation. Two patients with LPF died; one died of heart failure due to atrioventricular valve regurgitation after the third banding adjustment and one died after the subsequent bidirectional Glenn operation.

## Discussion

Band tightness of BPAB was considered effective if SaO_2_ decreased or if systolic blood pressure increased. Intraoperative echocardiography is preferred in many institutions because it is the only noninvasive method of evaluating band tightness on the right and left pulmonary arteries individually. Sakurai et al. arbitrarily adjusted band tightness to achieve systolic pulmonary artery blood flow velocities of 3.5 m/sec at the banding sites [2]. Kitahori et al. adjusted the band tightness to attain an intraoperative SaO_2_ of 75–85% with pulmonary artery flow velocities at the banding sites of at least 3.0 m/sec [3]. In searching the literature, however, we found no reports of identifying the indicator of appropriate band tightness in BPAB through statistical analyses.

In a report without statistical analysis, Kaneko et al. postulated that diastolic–to–systolic flow velocity at the banding site would be a better indicator of appropriate band tightness, according to their previous experience [4]. Our study was conducted to test this postulation. The results of part A indicated that VR is the best indicator of band tightness and that a VR between 0.45 and 0.58 is indicative of appropriate systemic–to–pulmonary blood flow balance. The results of part B showed that in relying on this indicator, the surgeons significantly reduced complications from excessive pulmonary blood flow. However, we did not reduce complications from insufficient pulmonary blood flow. The AUC of VR for HPF was larger than that for LPF, which implies that VR is less sensitive for LPF than for HPF. The results indicate that maintaining VR between 0.45 and 0.58 is crucial in preventing HPF. Conversely, we could not find a sensitive indicator for LPF. On the basis of the results of this study, we currently start BPAB with loose banding, sequentially tighten the bands until VR exceeds 0.45, and finish BPAB adjustment to prevent LPF.

### Limitation

Several factors other than band tightness might affect VR. If the diastolic blood pressure is low before the banding site, e.g., the semilunar valve regurgitation or obstructive patent ductus arteriosus in cases of duct dependent pulmonary circulation, the VR may be low due to a poor increase in diastolic blood flow. Difference in heart rate may affect VR due to the nadir point of velocity flow pattern shifting, so other measurement such as pressure half time need to be included in future studies.

With regard to echocardiographic indicators, other researchers may disagree with our use of the arithmetic mean of left and right measurements as a patient-specific value.

## Conclusion

In BPAB, we consider it is better to determine the degree of banding tightness with reference to the intraoperatively measured VR. The optimal range for VR is between 0.45 and 0.58. 

## Data Availability

Raw data were generated at National Center for Child Health and Development. The derived data supporting the findings of this study are available from the first author, Tetsuri Takei, upon reasonable request.
